# A method of cotton root segmentation based on edge devices

**DOI:** 10.3389/fpls.2023.1122833

**Published:** 2023-02-17

**Authors:** Qiushi Yu, Hui Tang, Lingxiao Zhu, Wenjie Zhang, Liantao Liu, Nan Wang

**Affiliations:** ^1^ College of Mechanical and Electrical Engineering, Hebei Agricultural University, Baoding, China; ^2^ College of Agronomy, Hebei Agricultural University, Baoding, China; ^3^ College of Modern Science And Technology, Hebei Agricultural University, Baoding, China

**Keywords:** *in situ* root, high-throughput phenotype, low-cost acquisition, semantic segmentation, edge equipment

## Abstract

The root is an important organ for plants to absorb water and nutrients. *In situ* root research method is an intuitive method to explore root phenotype and its change dynamics. At present, *in situ* root research, roots can be accurately extracted from *in situ* root images, but there are still problems such as low analysis efficiency, high acquisition cost, and difficult deployment of image acquisition devices outdoors. Therefore, this study designed a precise extraction method of *in situ* roots based on semantic segmentation model and edge device deployment. It initially proposes two data expansion methods, pixel by pixel and equal proportion, expand 100 original images to 1600 and 53193 respectively. It then presents an improved DeeplabV3+ root segmentation model based on CBAM and ASPP in series is designed, and the segmentation accuracy is 93.01%. The root phenotype parameters were verified through the Rhizo Vision Explorers platform, and the root length error was 0.669%, and the root diameter error was 1.003%. It afterwards designs a time-saving Fast prediction strategy. Compared with the Normal prediction strategy, the time consumption is reduced by 22.71% on GPU and 36.85% in raspberry pie. It ultimately deploys the model to Raspberry Pie, realizing the low-cost and portable root image acquisition and segmentation, which is conducive to outdoor deployment. In addition, the cost accounting is only $247. It takes 8 hours to perform image acquisition and segmentation tasks, and the power consumption is as low as 0.051kWh. In conclusion, the method proposed in this study has good performance in model accuracy, economic cost, energy consumption, etc. This paper realizes low-cost and high-precision segmentation of *in-situ* root based on edge equipment, which provides new insights for high-throughput field research and application of *in-situ* root.

## 1 Introduction

Roots play a crucial role in the absorption of water and nutrients by plants, affecting plant health, environmental adaptation and productivity (Hinsinger et al., 2011; Lynch and Wojciechowski, 2015; Paez-Garcia et al., 2015). Microroots (mainly composed of fine roots and root hairs) are the main executive parts of roots. The absorption of water and nutrients accounts for more than 75% of the total absorption of roots (Nielsen et al., 2001). The dynamic changes of their own morphological characteristics (Shan and Tao, 1992) significantly affect root function and plant growth. At present, root phenotype research focuses on the accurate identification of root architecture. However, the segmentation of plant roots from the cultivation environment is vulnerable to the impact of small and medium soil particles. At the same time, it is difficult to accurately segment the edges of roots and soil, which restricts the acquisition of accurate root images.

In order to solve the above problems, scholars at home and abroad have conducted a lot of relevant research. Obtaining high-resolution images of roots in soil is the basis for accurate identification of root configuration. Traditional root acquisition methods, such as root drilling, soil column method and profile method, consume materials and manpower. Problems such as damage to root configuration and loss of small root segments are easy to occur during extraction, which cannot meet the dynamic and accurate identification of root configuration, It has been replaced by *in situ* root observation (*in situ* cultivation method and *in situ* imaging method) (Xiao et al., 2020; Liu et al., 2020b). The root *in situ* imaging method originated from the micro root canal method (Bates, 1937; Cseresnyés et al., 2021; Rajurkar et al., 2022) It refers to identifying the root image contacting the glass tube wall by inserting a glass tube into the soil. However, its disadvantages lie in poor resolution (numerical value), slow acquisition speed (time), and low degree of automation. It is difficult to achieve batch synchronization and real-time acquisition of the original root image. In addition, X-ray tomography (XCT) and nuclear magnetic resonance imaging (MRI) commonly used in medicine also provide new methods and means for the acquisition of *in situ* root images (Jahnke et al., 2009). XCT scans the root image by using the characteristics of different attenuation degrees of X-ray passing through the soil and root, and finally obtains the root image (Park et al., 2020; Scotson et al., 2021; Ferreira et al., 2022). MRI is a modern tomographic imaging technology, which mainly transmits radio frequency electromagnetic waves to obtain the MRI information of different positions of objects in the magnetic field to generate images, and uses computers to reconstruct the internal images of objects(Borisjuk et al., 2012). It has also been applied in root research (Schneider et al., 2020; Horn et al., 2021; Pflugfelder et al., 2021). However, there are still drawbacks to the above two technologies. Among them, XCT imaging takes a long time to acquire, while MRI is more suitable for acquiring large roots. Neither of them can recognize that the diameter is less than 400 μ M (Metzner et al., 2015), and both technologies have disadvantages such as high equipment cost and vulnerability to soil environment interference.

Digital equipment imaging method can dynamically collect high-resolution *in situ* root images without changing the soil environment and affecting the root growth state, which is conducive to improving the efficiency of root configuration segmentation and quantitative analysis (Hammac et al., 2011). In recent years, it has been widely reported that simple cultivation devices combined with digital equipment (smart phones, scanners, digital cameras) are used to obtain root images (Mohamed et al., 2017; Nakahata and Osawa, 2017; Nahar and Pan, 2019).

On the basis of accurately obtaining high-resolution *in situ* root images, accurate and efficient root configuration recognition is a thorny problem in current root phenotype research (Lynch, 2013). The traditional image processing methods for root recognition include traditional manual description, semi-automatic interactive recognition and automatic threshold segmentation. The manual description method has the problems of low recognition efficiency, large workload and high result error (Abramoff et al., 2004; Le Bot et al., 2010). The semi-automatic method is based on visual observation and image recognition through auxiliary software. Although semi-automatic interaction can achieve high accuracy, it is too dependent on the subjective ability of observers to distinguish roots and their own experience. The segmentation of a single complex root image takes a long time, and the efficiency is too low to achieve high flux *in situ* root image analysis. Although the fully automatic threshold method improves the efficiency of root identification, such as DIRT, GiaRoots, IJ Rhizo and EZ Rhizo can provide statistical information such as root diameter, height and density (Galkovskyi et al., 2012; Pierret et al., 2013; Das et al., 2015). However, it is difficult to eliminate the noise interference of soil background, and there are errors in root morphology identification. And most of the research focuses on obtaining the more extensive root parameters such as structure, length, diameter, etc. It is difficult to excavate more detailed morphological characteristics of micro roots.

Compared with traditional methods, root recognition based on deep learning is easier to mine multi-level characteristics of the target, and occupies a dominant position in the current root phenotype research. For example, the SegRoot platform (Wang et al., 2019) can mine multi-scale features of root images through the improved SegNet network (Badrinarayanan et al., 2017), but under fitting may occur in some cases. The ITErRoot network (Seidenthal et al., 2022) has achieved good results in root segmentation by stacking the encoding, decoding layer and residual structure of the U-shaped structure many times, but its network is too bloated and requires a high training platform. The RootNav2.0 system (Yasrab et al., 2019) is based on the encoder decoder CNN architecture and replaces the previous semi-automatic feature extraction RootNav system (Pound et al., 2013) with a multitask convolutional neural network architecture. It does not require user interaction to accurately extract the root structure, and the speed is increased by nearly 10 times, but it needs to be carried out when the root is fully visible. Through the improved UNet structure, the FaRIA platform (Narisetti et al., 2021) divides the large resolution image into 256 x 256 small images for prediction, and realizes the batch prediction of root images. The RootPainter platform (Smith et al., 2022) includes semi-automatic and fully automatic methods. The former allows users to subjectively correct each segmented image, and the model can learn from the assigned correction, reducing the segmentation time with the segmentation process; the latter is more suitable for processing large data sets.

Edge devices include raspberry pie series developed by Raspberry Pi Foundation, jetson series developed by Nvidia Company, and orange pie series developed by Xunlong Software Company. Among them, raspberry pie is increasingly used as a low-cost, high-throughput solution for plant phenotype analysis (Jolles, 2021). For example, the “Do It Yourself” phenotyping system (Dobrescu et al., 2017) uses raspberry pie control cameras to achieve batch plant image acquisition, PYM (Valle et al., 2017) uses raspberry pie control infrared cameras to perform phenotype analysis on plant leaves, and Greenotyper (Tausen et al., 2020) uses raspberry pie control cameras and deploys depth learning to monitor plant positions. However, most of these platforms are used for phenotypic analysis of plant parts on the ground, lacking of research cases on plant underground roots. At present, mainstream platforms for root identification, such as RhizoVision Crow (Seethepalli et al., 2020), are based on desktop development and do not support deploying models to Raspberry pie.

It has been reported that the RhizoPot platform was developed by our research team in the early stage (Zhao et al., 2022) can realize high-resolution, non-destructive real-time acquisition of *in-situ* root images. In addition, the team has designed a cotton plant root segmentation method based on DeeplabV3+ and proposed the improvement strategy of the model for the research on root segmentation methods (Shen et al., 2020; Jia et al., 2021). However, the previous studies were all indoor platform development, and the equipment cost was high, lacking the exploration of portable equipment in outdoor environment. Therefore, based on the previous research, this paper designs a data augmentation scheme to expand the data set; DeeplabV3+ model is modified to connect CBAM attention mechanism with ASPP spatial pyramid pooling; The prediction strategy is modified to make it more suitable for edge devices; Deploy to Raspberry pie, and design the method of field experiment. The purpose of this paper is to design a low-cost, high-throughput *in situ* root precise identification technology by replacing the traditional GPU analysis platform with raspberry pie, and explore the possibility of its application in outdoor environment.

## 2 Materials and methods

### 2.1 Image collection

**Figure 1:**
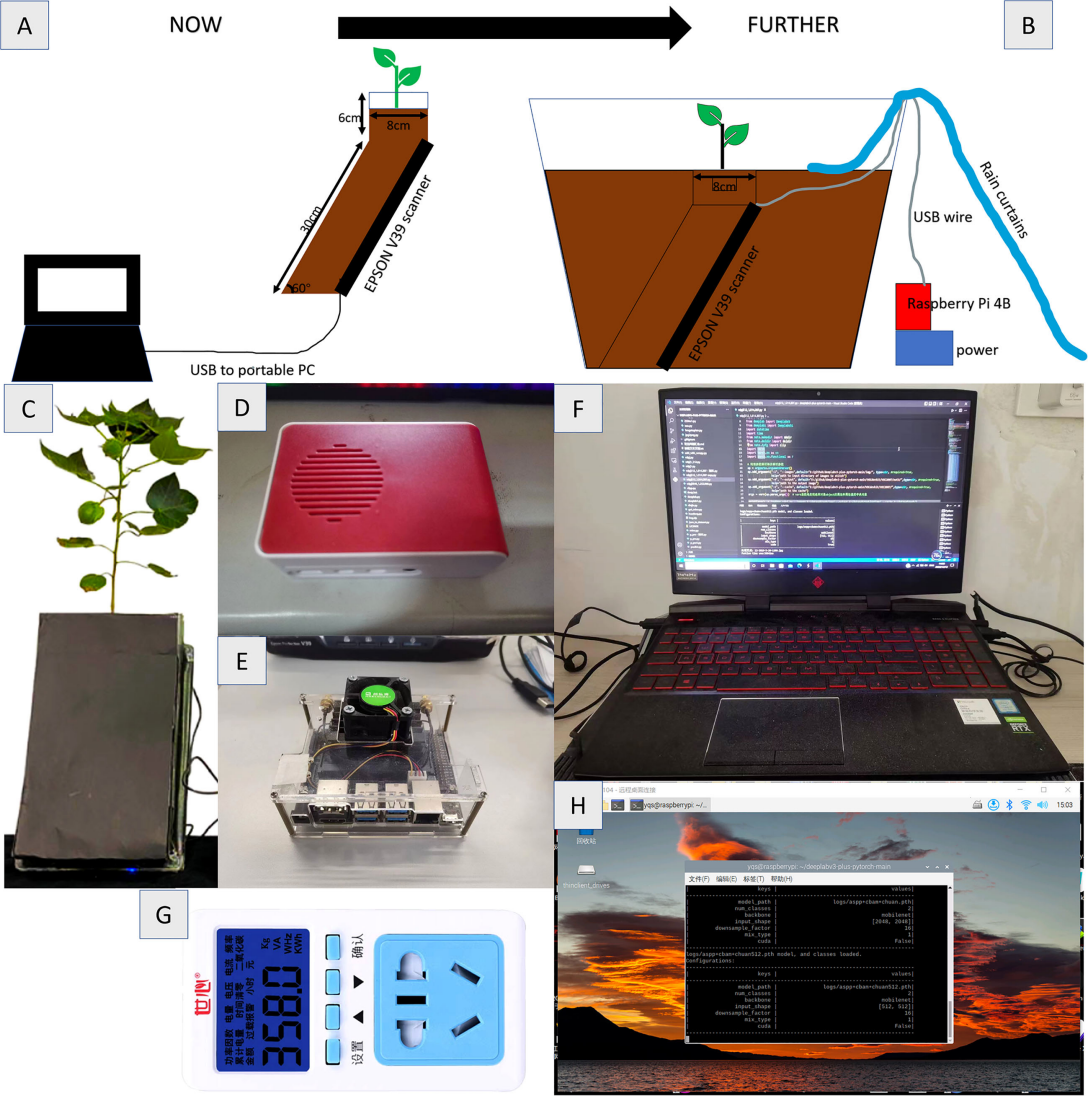
Root collection equipment and method **(A)** Schematic diagram of RhizPot **(B)** Schematic diagram of field test **(C)** RhizoPot **(D)** Raspberry Pie 4B **(E)** Jetson Nano **(F)** RTX2060 Notebook **(G)** Power Detector **(H)** Raspberry Pie Remote Desktop.

This experiment was conducted in the experimental station of Hebei Agricultural University in Baoding, Hebei Province (38.85°N, 115.30°E) in 2021. The climate of the experimental site was mild. Use Epson scanner V39 (Epson lnc., Suwa shi, Nagano, Japan) to scan root images in batches. The resolution of the collected images is set to 1200dpi and the saved format is bmp. The experimental schematic diagram and equipment are shown in **Figures 1A, C** respectively. **Figure 1B** shows the prospect of field experiments. **Figures 1D–H** are the relevant experimental equipment.

This paper filters and classifies the collected image set, removes incomplete and fuzzy images, and finally retains 125 complete and clear cotton roots *in situ* images, randomly selects 100 of them for network training, and the ratio of training set to verification set is 9:1. According to the image data expansion method proposed in this paper, 47873 and 1600 training set images and 5320 and 160 verification set images are finally obtained. The remaining 25 images are used as a test set to evaluate the network performance.

The image annotation is completed by an experienced agronomist using the Adobe Photoshop CC (Adobe Inc., San Jose, CA, United States) lasso tool. All pixels considered as roots are marked white and saved in a new layer. Finally, the remaining pixels are marked black. The resolution of the annotation image is 10200 pixels x 14039 pixels, and the annotation time of a single image is about 4.5 hours.

### 2.2 Data augmentation

The dataset format required for training DeeplabV3+ is jpg, and the image data set needs to be converted from bmp to jpg. In this paper, two image data augmentation methods are designed. In method 1, the training images are divided according to the resolution of 512 pixels x 512 pixels to ensure that the training can be carried out by resolution. At this time, the input image data set is expanded to 53193, and the training set and verification set are 47873 and 5320, respectively.

**Figure 2:**
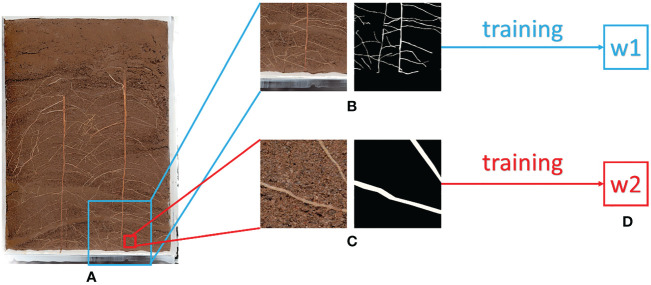
Illustration of Equal Proportion Dataset and Pixel by Pixel Dataset **(A)** Original Image **(B)** Equal Proportion Dataset **(C)** Pixel by Pixel Dataset **(D)** After Training, w1 is the weight trained by the Equal Proportion Dataset, and w2 is the weight trained by the Pixel by Pixel Dataset.

In method 2, the training input image is segmented according to the size ratio. To ensure accurate prediction of the original image, the input resolution of the whole image is set to 2048 pixels x 2048 pixels. During training, the input resolution needs to be kept at 512 pixels x 512 pixels. The ratio of training resolution to prediction resolution is 1: 16. Therefore, the image is reduced to 1/16 of the original one to ensure that the prediction of the whole image has a fixed size ratio. The annotation image also needs to go through the same processing process, and the final training set based on equal proportion is 1600 pieces, and the verification set is 160 pieces. After training, the w1 weight based on the equal proportion method and the w2 weight based on the pixel by pixel method are obtained **Figure 2** shows the differences between the two methods. 

### 2.3 Segmentation model

#### 2.3.1 Model comparison

**Figure 3:**
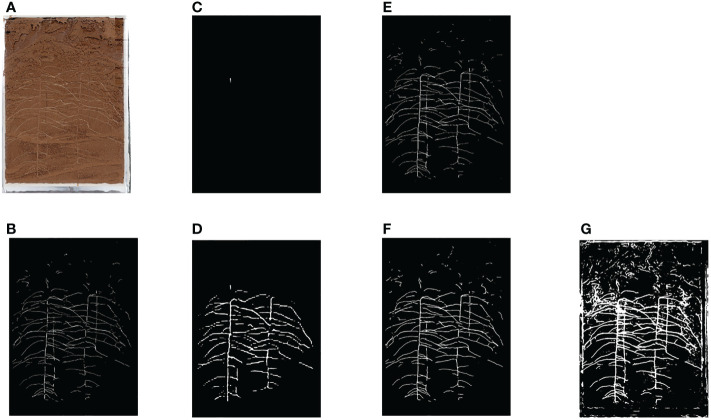
Segmentation Results of Various Network Roots **(A)** Original Image **(B)** Ground Truth **(C)** PSPNet **(D)** HRNet **(E)** UNet **(F)** DeeplabV3+(MobilenetV2) **(G)** DeeplabV3+(Xception).

The root data set used in this paper is selected in turn to compare DeeplabV3+(Chen et al., 2018), PSPNet (Zhao et al., 2017), HRNet (Sun et al., 2019) and UNet (Ronneberger et al., 2015). The image segmentation results are shown in **Figure 3** and the experimental data are shown in Research 1 of **Table 1**.

**Table 1:** Research 1: Performance of each network partition.

Research	Network name	mIoU(%)	mPA(%)	mPrecision(%)	Precision root(%)	Precision background(%)	Recall(%)	GPU mtime(min)	Raspberry Pie mtime(min)
Research 1	PSPNet	51.95	63.8	53.93	8.43	99.43	63.8	NA	NA
	HRNet	60.23	67.94	69.09	40.12	98.06	67.94	NA	NA
	DeeplabV3+	67.18	70.25	91.26	86.15	96.36	70.25	NA	NA
	(Xception)							NA	NA
	UNet	**85.06**	**90.8**	91.83	84.2	**99.46**	**90.8**	NA	NA
	DeeplabV3+	75.17	78.22	**92.9**	**87.75**	98.05	78.22	NA	NA
	(MobilenetV2)							NA	NA
Research 2	Series	74.53	77.49	**93.01**	**88.09**	97.92	77.49	NA	NA
	Parallel	**75.77**	**79.16**	92.25	86.28	**98.23**	**79.16**	NA	NA
Research 3	Fast	85.45	**91.96**	91.19	**83.37**	99.55	**91.96**	**0.599**	**26.01**
	Normal	**85.64**	91.94	**91.46**	82.82	99.55	91.94	0.775	41.19

Research 2: Performance comparison of two methods to improve DeeplabV3+. Research 3: Performance of Fast segmentation and Normal segmentation on raspberry pie 4B and GPU platforms.NA, Not Applicable. The optimal values are written in bold font.

From the segmentation effect of root image (**Figure 3**), it can be seen that DeeplabV3+(MobilenetV2) and UNet have the best segmentation effect, while DeeplabV3+(Xception) has obvious under fitting phenomenon. The segmentation effect of HRNet and PSPNet is very poor, and the model is not suitable for root segmentation.

According to the prediction results of root image (Research 1 of **Table 1**), DeeplabV3+(MobilenetV2) has the best effect, followed by UNet, DeeplabV3+(Xception), HRNet and PSPNet.

Therefore, based on the previous experimental results, this paper designs a backbone network based on DeeplabV3+ model and MobilenetV2 to train and predict the root image.

#### 2.3.2 Model improvement

At present, the attention mechanism can significantly improve the model feature extraction ability and can be embedded in most mainstream networks without significantly increasing the model parameters and computation. Attention module includes channel attention module, space attention module, time attention module and branch attention module, and mixed attention mechanism: channel space attention mechanism and space time attention mechanism. CBAM (Woo et al., 2018) is an attention module in the channel spatial attention mechanism. That is, the image first goes through the channel attention mechanism (CAM) to solve the problem of “what to pay attention to”, then goes through the spatial attention mechanism (SAM) to solve the problem of “where to pay attention to”, and finally integrates with the original feature map to form a new feature map that emphasizes the channel and spatial features. In addition, CBAM is a lightweight attention mechanism that can be seamlessly integrated into any neural network without module overhead.

**Figure 4:**
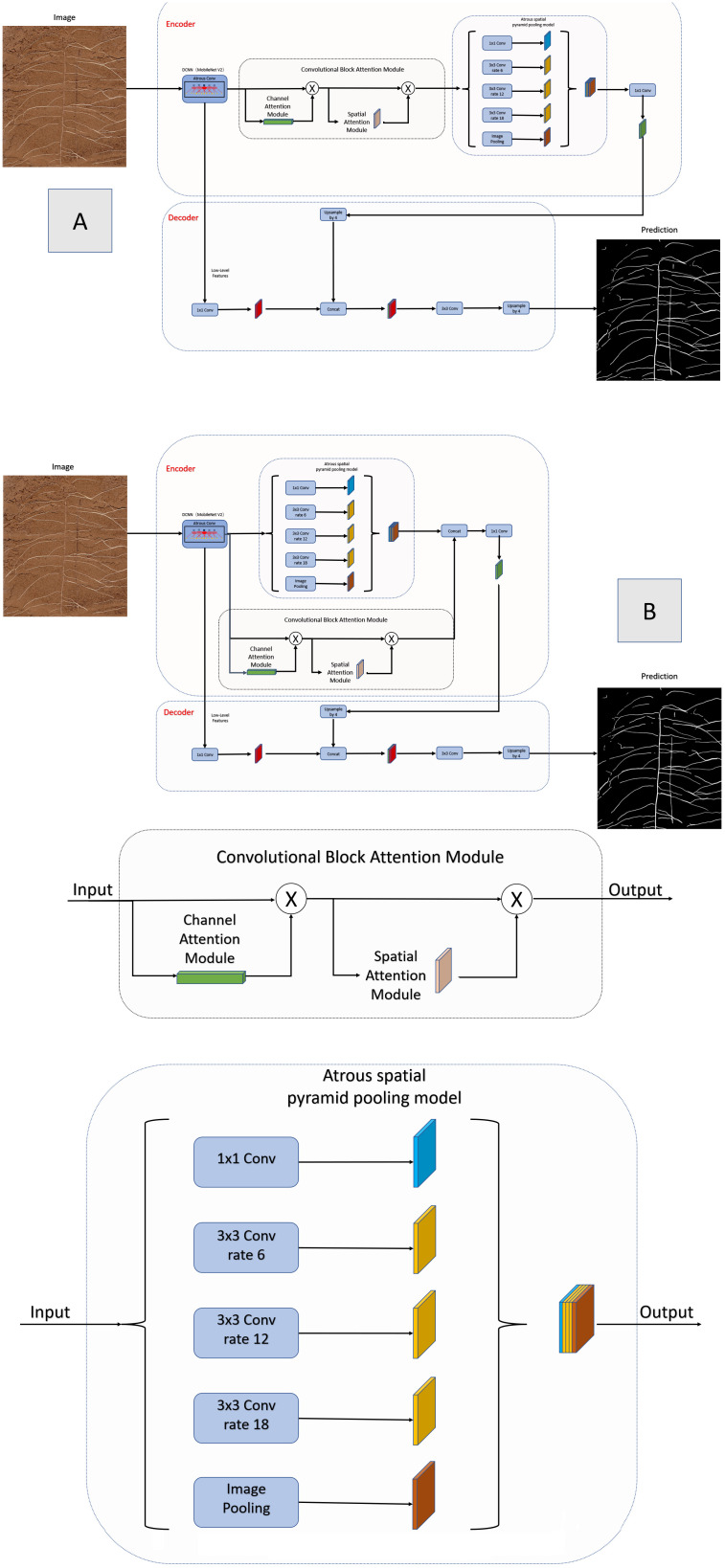
Improved DeeplabV3+ network structure **(A)** Series improvement **(B)** Parallel improvement.

**Figure 5:**
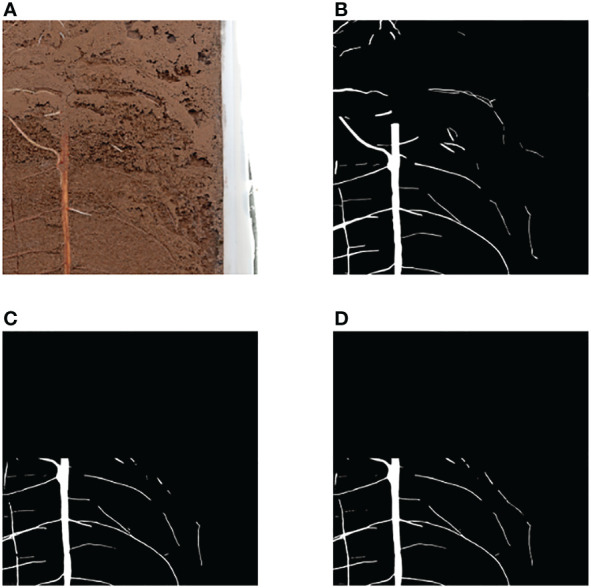
Serial and Parallel Segmentation Results **(A)** Original Image **(B)** Ground Truth **(C)** Parallel Segmentation Image **(D)** Parallel Segmentation Image.

The DeeplabV3+ model designed in this paper uses the ASPP structure in the encoder part, which contains three parallel hole convolutions with expansion rates of 6, 12 and 18, which can provide a larger receiving field and capture more context information. On this basis, inspired by the deployment of the dual attention mechanism to the DeeplabV3+ network (Liu et al., 2020a). This paper tests two methods of CBAM attention mechanism deployment to the DeeplabV3+ network, namely, the series connection and parallel connection of CBAM and ASPP. The network structures are shown in **Figures 4A, B** respectively. Both methods use the pre training weight of the backbone network to iterate for 100 times before performance testing. **Figure 5** compares the segmented images of the two methods, and the performance comparison is shown in Research 2 of **Table 1**. The results show that the CBAM attention mechanism in series with ASPP is better than the parallel operation.

### 2.4 Predictive policy

**Figure 6:**
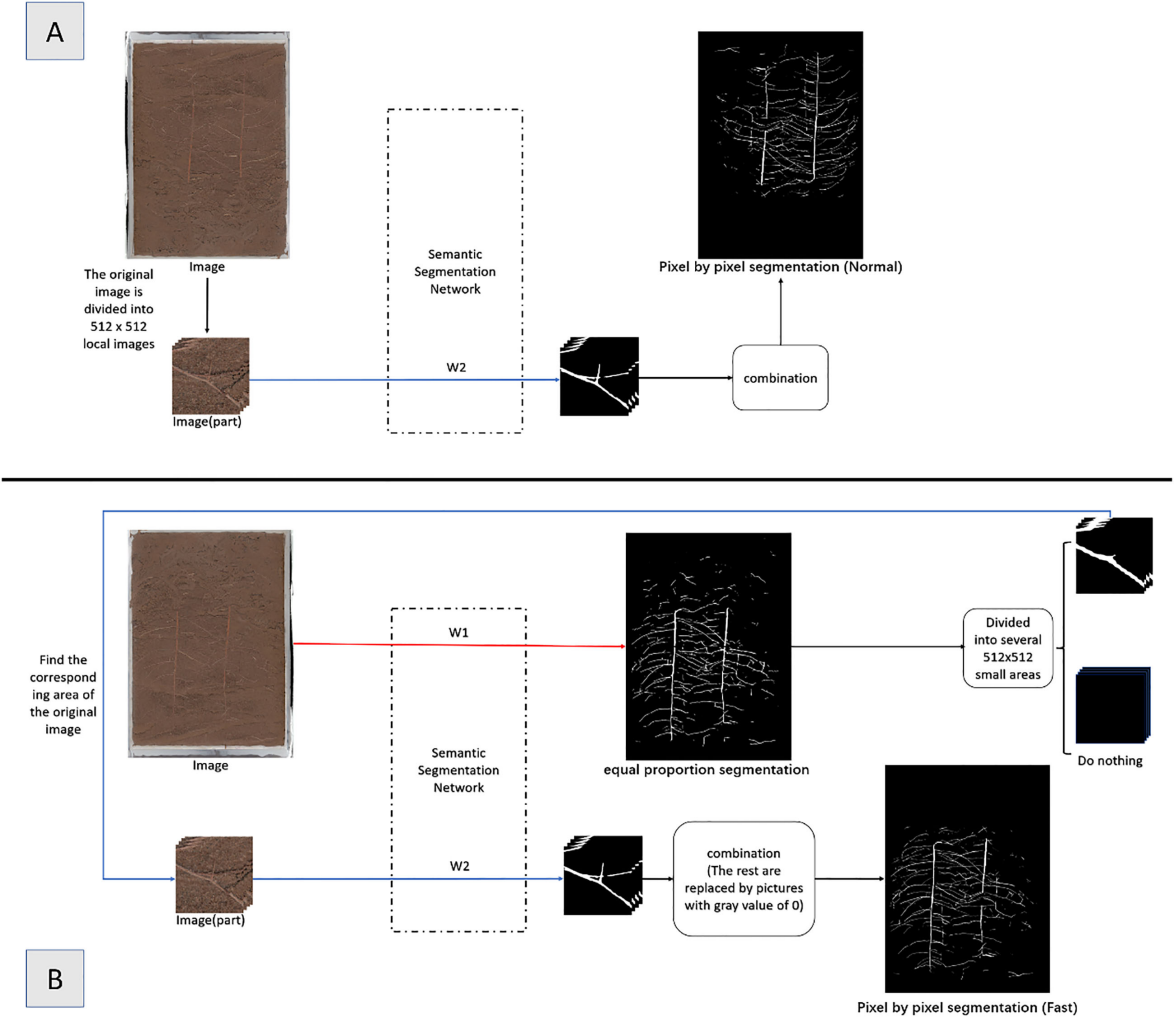
Division Method Diagram **(A)** Normal Division Method **(B)** Fast Division Method.

In the early stage of this paper, the traditional conventional strategy (Normal) of segmentation followed by splicing was tested. By dividing the original image to a specified size, network prediction was performed, and then the prediction results were spliced to obtain a complete segmentation result. The processing process is shown in **Figure 6A**. The test results show that although the prediction accuracy of this method is high, it takes a long time to predict after deployment to raspberry pie. The shared time of the test set image prediction process is up to 17 hours and 10 minutes. Therefore, this paper proposes an improved fast splicing and segmentation strategy (Fast).

**Figure 7:**
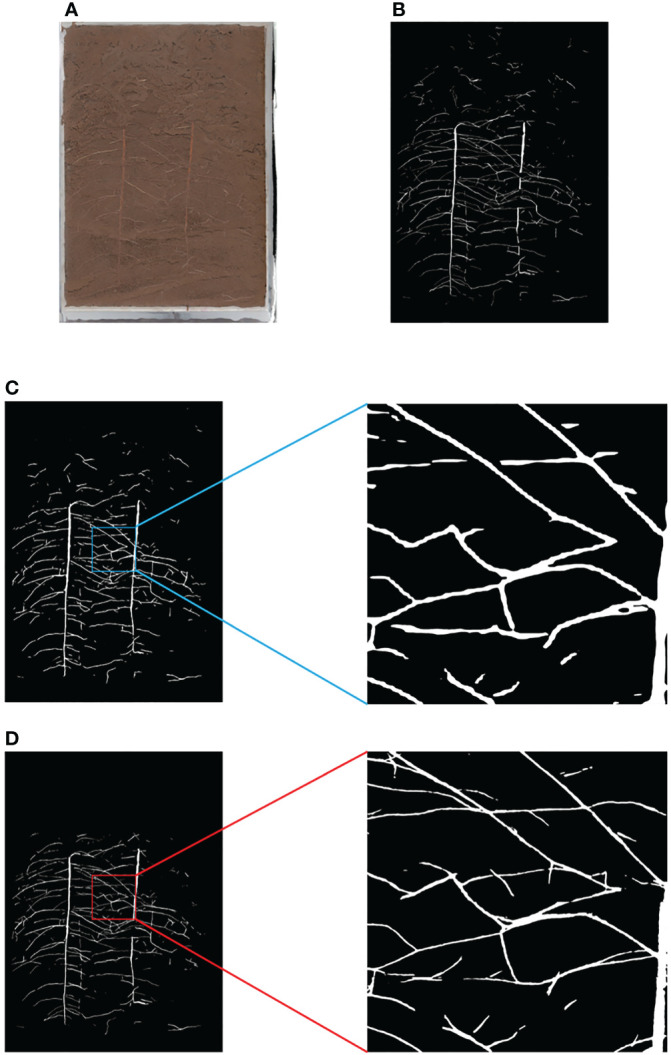
Comparison of Equal Proportion Image Segmentation and Pixel by Pixel Image Segmentation **(A)** Original Image **(B)** Ground Truth **(C)** Results and Details of Equal Proportion Segmentation **(D)** Results and Details of Pixel by Pixel Segmentation.

The improved Fast method is shown in **Figure 6B**. Based on the results of the full image processing of the equal proportion segmentation (**Figure 7C**), the region is divided into foreground and background, where the foreground is the part of the region that contains roots, and the background is the part of the region that does not contain roots. For the foreground part, the region corresponding to the original image is put into the network for segmentation; for the background part, a RGB image with all gray values of 0 is directly used to replace it. Finally, combine the two into a complete segmented image (**Figure 7D**).

The results show that this method can save 22.71% of the time cost on GPU on average, and the segmentation accuracy only decreases 0.55% year on year, as shown in Research 3 of **Table 1**. Later deployed in the raspberry pie terminal, the time cost of a single picture can be saved by 36.85%.

### 2.5 Raspberry pie deployment

The model of the edge device selected in this article is Raspberry Pi Foundation (Cambs, United Kingdom) 4B, which contains 8G of memory, plus 32G of memory card. Raspberry Pie is an ARM based microcomputer motherboard. SD/MicroSD card is used as the memory hard disk. There are 1/2/4 USB interfaces and a 10/100 Ethernet interface (Type A has no network interface) around the card motherboard. It can connect the keyboard, mouse and network cable. It also has a TV output interface for video analog signals and an HDMI high-definition video output interface.

#### 2.5.1 Installation of raspberry pie system

The raspberry pie system selects the raspberry pie official 64 bit system image (Raspberry Pi), sets SSH, WIFI, language and time zone through the official burning software, and then burns it into the 32G memory card. After startup, connect to Raspberry Pie *via* MobaXterm to configure corresponding functions.

#### 2.5.2 Raspberry pie deployment batch splitter

**Figure 8:**
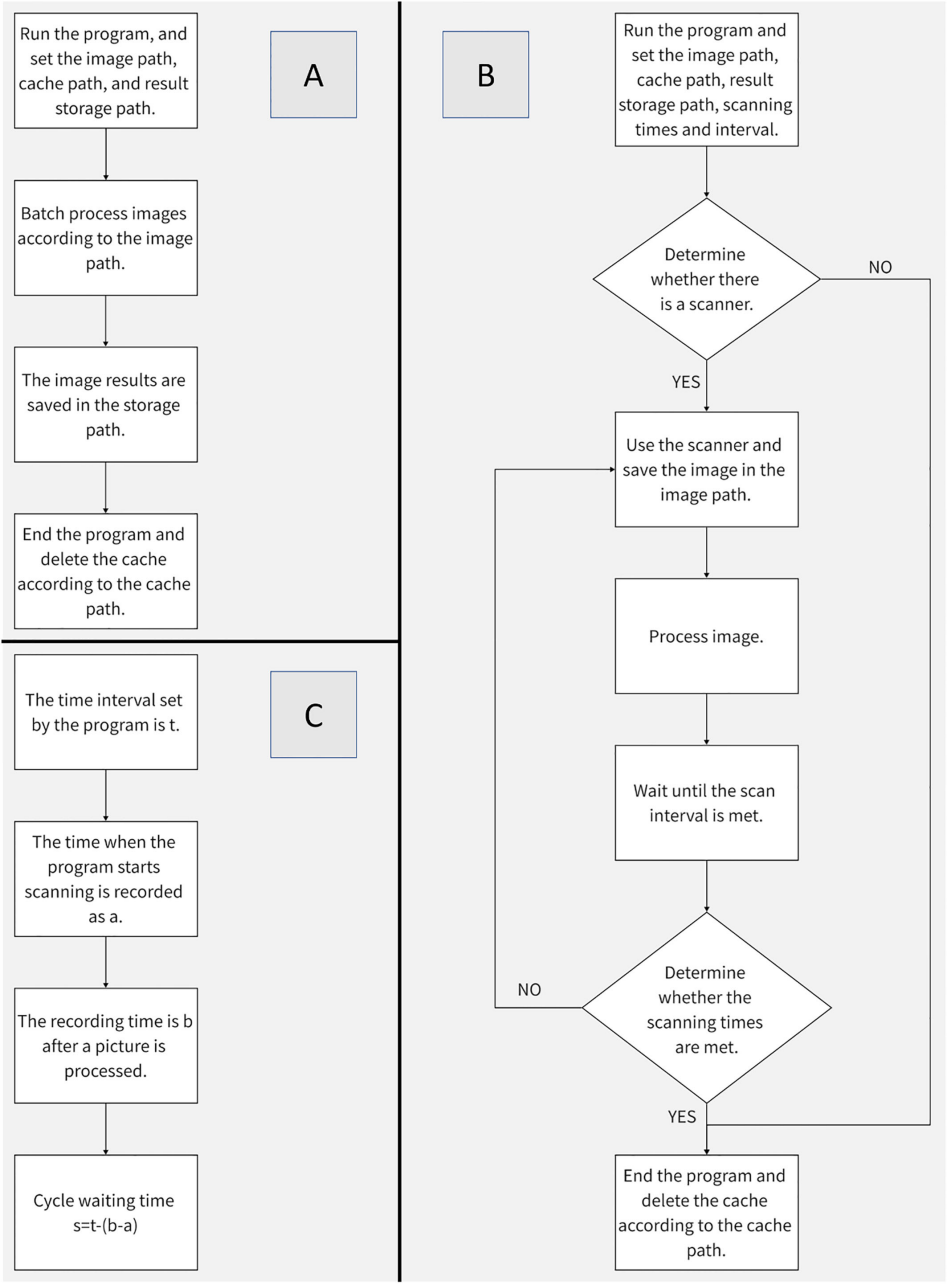
Procedure Flow Chart **(A)** Batch Segmentation Procedure Flow **(B)** Image Acquisition and Segmentation Procedure Flow **(C)** Method of Image Acquisition Interval.

Deploy the batch splitting program to Raspberry Pie 4B, and its settable parameters include picture address, cache address and target address. Its operation process is shown in **Figure 8A**.

#### 2.5.3 Raspberry pie participates in image collection and segmentation

In order to give full play to the low power consumption advantage of Raspberry Pie 4B, this paper combines the image segmentation program with image acquisition, and can set parameters including scanner name, acquisition quantity, interval time, image storage address, cache address, and target address (the default setting of the scanner is dpi=1200, and the color mode is color). The operation process is shown in **Figure 8B**. Compared with batch segmentation, continuous collection and segmentation can better reflect the advantages of raspberry pie 4B. The method of timing acquisition is shown in **Figure 8C**.

**Figure 9:**
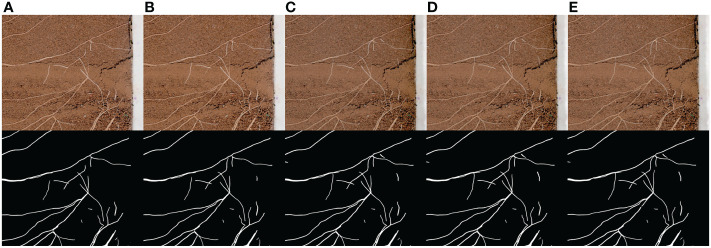
Continuous acquisition and segmentation results. **(A–E)** are the root image and network segmentation result collected continuously at a time interval of 24 hours.

The collection results are shown in **Figure 9**. The collection interval is 24 hours and the collection time is 19:00 every day. In order to show the growth process of root more clearly, the original image is cropped, and the change trend of root can be clearly observed in the image after network segmentation.

## 3 Results

### 3.1 Model evaluation

#### 3.1.1 Model selection

This paper compares the performance of PSPNet, HRNet, UNet and DeeplabV3+ depth learning models in cotton root image segmentation. The test results show the comprehensive performance of DeeplabV3+ > UNet > HRNet > PSPNet.

In DeeplabV3+, compare the backbone networks MobilenetV2 and Xception, and see Research 1 of **Table 1** for performance comparison when the backbone network pre training weight is used to train iterations for 100 times. The effect of MobilenetV2 is better than that of Xoption because MobilenetV2 is lighter, the network model is smaller, the number of iterations required is less, and the prediction time is shorter. The Xception network model is larger, which is not conducive to deployment to edge devices.

#### 3.1.2 Improvement results

This paper compares CBAM with ASPP in parallel and in series. **Figure 4** for network structure and Research 2 of **Table 1** for segmentation performance. The results show that in terms of segmentation accuracy, the method of CBAM in series with ASPP is the best, DeeplabV3+ is the second, and the method of CBAM in parallel with ASPP is the second.

In addition, the prediction accuracy of the improved DeeplabV3+ network is 90.15%, higher than DeeplabV3+, HRNet and PSPNet, and slightly lower than Unet. However, because the input and output sizes of the Unet network are inconsistent, resulting in burrs on the edge of the output prediction image. At the same time, the cost of the Unet network is slightly higher. If the Unet network is directly deployed to the Raspberry Pie 4B, it will not be able to segment the high-resolution root image due to memory limitations. Therefore, this paper does not use Unet as the root segmentation network.

The test results show that the improved DeeplabV3+ network can be directly deployed to the raspberry pie 4B, without pruning, and runs well with stability and reliability.

### 3.2 Performance evaluation

#### 3.2.1 Predictive performance

After the network is deployed to Raspberry Pie, the average partition time of the test set is about 41.19 minutes with the Normal strategy, while the average partition time of the test set is about 26.01 minutes with the Fast strategy, a year-on-year decrease of 15.18 minutes. It takes about 9 minutes to segment images with sparse roots and 33 minutes to segment images with dense roots. Compare the segmented image with the labeled image, and the performance indicators are shown in Research 3 of **Table 1**.

Therefore, compared with the Normal strategy, the Fast strategy runs 36.85% faster in raspberry pie 4B on average, but the prediction accuracy is only 0.34% lower. The experimental results show that the Fast segmentation strategy proposed in this paper can replace the Normal strategy to a certain extent.

#### 3.2.2 Prediction accuracy

In addition, this paper also uses the open-source Rhizo Vision Explorers platform (Seethepalli et al., 2021) to analyze the phenotypic data of segmented root images, mainly comparing the differences between the result images and the labeled images in root length and diameter. See **Table 2** for the comparison results. The results show that the error of root phenotypic parameters such as root length and diameter obtained by Normal strategy and Fast strategy compared with the original labeled image is acceptable. However, there is a big error between the root length, diameter and the actual value of the result image obtained by the equal proportion segmentation method.

**Table 2:** Comparison of equal proportion segmentation, pixel by pixel segmentation and ground truth root system.

Name	Total root length (pt)	Root length error (%)	Total root diameter (pt)	Root diameter error (%)
Ground Truth	5576876	NA	534.835	NA
Equal Proportion	6057449	8.617	837.174	56.529
Pixel by Pixel (Normal)	5659371	1.479	538.775	**0.737**
Pixel by Pixel (Fast)	5614186	**0.669**	540.198	1.003

NA, Not Applicable. The optimal values are written in bold font.

### 3.3 GPU verification

The advantages of edge devices replacing host devices lie in high portability and low power consumption. In this paper, the Raspberry pie 4B with 8G memory and HP Shadow Genie 5 (i7-9750h+RTX2060+16G memory) notebook computers are used, and the power supply is connected through the P06S-10 power detector to compare the power consumption of the two in the continuous 8-hour image acquisition and segmentation. The acquisition rate is 1 piece per hour, 8 pieces in total are collected, and the image is segmented in the acquisition window period. The experimental equipment is shown in **Figure 1**, and the results are shown in **Table 3**.

**Table 3:** Comparison between Raspberry Pie 4B and RTX2060 Notebook for Collection and Batch Segmentation.

Method	Platform	Average split time (h)	Collection time (h)	Total time (h)	Power consumption (kWh)
Collection Segmentation	RTX2060	NA	8	8.008	1.281
	Raspberry Pie 4B	NA	8	8.496	0.051
Batch Segmentation	RTX2060	0.017	NA	0.433	0.073
	Raspberry Pie 4B	0.434	NA	10.338	0.086

NA, Not Applicable.

#### 3.3.1 Power consumption verification

The power consumption of Raspberry Pie 4B is much lower than that of RTX2060 platform when collecting and segmenting the same image. At the same time, when the time interval between two root image scans exceeds 30 minutes, Raspberry pie 4B has the ability to segment within the scanning interval. The time used will not increase, but the power consumption will be greatly reduced. Therefore, it is proved that if the scanning interval is allowed, the edge devices can deploy root segmentation networks to completely replace the high cost and high energy consumption GPU analysis platform.

#### 3.3.2 Time verification

In this paper, the Fast policy and the Normal policy are deployed on the GPU platform for comparison. The results show that the Fast policy is 22.71% faster than the ordinary policy on average, which verifies the conclusion that the Fast policy takes less time than the Normal policy.

#### 3.3.3 Result validation

In this paper, we also carried out a comparative experiment of raspberry pie and GPU batch segmentation of root images simultaneously. Both of them used 25 test set images for continuous segmentation test, with the same weight. The results are shown in **Table 3**, which verify that the raspberry pie is completely consistent with the GPU in terms of segmentation accuracy. However, due to the limitation of 4B computing power of Raspberry pie, the total power consumption is slightly higher than that of the graphics card.

### 3.4 Cost evaluation

Theoretically, semantic segmentation on the Jetson Nano with GPU in this experiment will accelerate, but since the memory of the Jetson Nano is only 4G, virtual memory needs to be added for network deployment. **Table 4** records the reference price of the equipment used in the experiment. Based on the data provided by the RhizoPot platform (Zhao et al., 2022), the cost of using the RTX2060 laptop with the Tensor core to control the RhizoPot platform is approximately $1480. Compared with using the Jetson Nano to control the RhizoPot platform, the cost is reduced to $301, but the cost of using the raspberry pie 4B is only $247 (excluding the power detector). Considering the cost, the performance price ratio of raspberry pie 4B or Jetson Nano is much higher than others. Compared with the segmentation performance of raspberry pie 4B and Jetson Nano, finally, this paper selects raspberry pie 4B as the edge device for deploying root image acquisition and segmentation.

**Table 4:** Reference Price of Equipment Used in the Experiment.

Name	Type	Price	Other
Raspberry Pi	Raspberry Pi 4B 8G	$35	
16G TransFLash card	Kingston	$5	
RhizoPot platform		$207	Including scanner, USB cable, acrylic plate and glass sealant
Jetson Nano		$89	
RTX2060 notebook	RTX2060 + 16G Memory	$1,268	
Power detector	Worldliness	$4	
Total		$1,608	

## 4 Discussion

### 4.1 Basis for model improvement

The previous results show that both CBAM and ASPP can improve the cotton image segmentation accuracy in series or in parallel, but the series method is better than the parallel method. The author believes that:

First, data processing samples are differentiated. The references are for remote sensing image data sets. This paper uses cotton root data sets. The characteristics of the two images are different. The remote sensing image is characterized by the buildings, farmland and other objects collected are basically square, while the cotton root image is irregular, similar to human blood vessels. In addition, remote sensing image segmentation usually faces multi category problems, and cotton root segmentation mainly focuses on two category problems.

Secondly, the series and parallel extraction features are differentiated. The advantage of concatenation is that after CBAM extracts multi-channel attention mechanism features, ASPP is used to sample multi-scale convolution kernel, which can more effectively extract root feature vectors in space and time. In the parallel connection method, on the premise that MobilenetV2 is used as the backbone network, the number of channels output by CBAM is 320, and the number of channels output by ASPP is 256, that is, the number of channels entering feature fusion is 576. However, in order to keep consistent with the number of channels in the network decoding part, the number of channels output by feature fusion can only be 256. Therefore, the rise of input feature fusion dimension leads to higher difficulty of classification, thus reducing the accuracy of the network.

Finally, for the optimization of the tandem method, residual structure, convolution block and pooling layer can be introduced into the attention mechanism module in the later stage to mine the characteristics of attention mechanism at multiple scales.

### 4.2 Forecast strategy validation

At the beginning of this experiment, the image is similar to pixel merging processing, that is, using the super pixel method (Ren et al., 2019), SLIC super pixel segmentation algorithm is introduced in the prediction, and the input image is divided into a super pixel image. The traditional DeeplabV3+ output operation of these super pixel image regions is used to obtain an accurate segmentation image. However, in actual processing, the SLIC is used to block the image, and the operation of block by block super pixel area will greatly increase the prediction time. At the same time, when the prediction image is more complex and fine, the quality of the output image will be seriously affected. At the same time, the high resolution image also limits the strategy of semantic segmentation.

There are two common methods for semantic segmentation of high-resolution images. The first method is to down sample the image and put it into the network for prediction, and then up sample the results, so that the image processing speed is fast and the context information will not be lost. However, the results of root phenotype analysis showed that the root length and diameter predicted by this method were larger than expected. The second is to use the sliding window operation to divide the image into the same area with a specified size and about 20% reserved, input the network prediction to obtain local results, and then complete the stitching of image feature points. This method has a good degree of detail and retains the context information, but it has high time cost and is not friendly to edge devices.

The fast splicing and segmentation strategy (Fast) proposed in this paper lacks context information, so the prediction effect of scattering is poor. The equal proportion segmentation part of this method actually belongs to the first kind of common segmentation method, which contains all the context information of the image. However, as shown in **Figure 7**, the image processed by this method lacks details, and the root length and diameter errors are too large after root phenotype analysis. Therefore, this method only uses it as a pedal to save the computing time of edge devices.

### 4.3 Edge device comparison

Compare the performance of Jetson Nano and raspberry pie 4B with similar prices in *in situ* root segmentation. See **Table 5** for the results. In the experiment, 25 cotton root images (from the test set) were selected for continuous and equal proportion segmentation, and the network weights used by Jetson Nano and Raspberry Pie 4B were consistent. Due to memory limitations, the Jetson Nano cannot segment 1200dpi images with a resolution of 10200 pixels x 14039 pixels. It can only compare 300dpi root images. In terms of program startup, when using cuda, the Jetson Nano can only segment up to three images consecutively, and then it will report an error that the timer has timed out. When cuda is not used, the segmentation time of the Jetson Nano for 300dpi images is much longer than that of the raspberry pie 4B.

**Table 5:** Comparison between raspberry pie 4B and jetson nano (the processing method used for comparison is equal proportion segmentation, 25 sheets).

Name	Split time(300DPI)(ms)	Split time(600DPI)(ms)	Split time(1200DPI)(ms)	Power consumption (300DPI)(kWh)	Power consumption (600DPI)(kWh)	Power consumption (1200DPI)(kWh)
Raspberry Pi 4B	1747842ms	1834684ms	2060141ms	0.004	0.004	0.005
Jetson nano	CUDA error	CUDA error	Memory error	NA	NA	NA
(CUDA)						
Jetson nano	17960733ms	18582792ms	Memory error	0.021	0.031	NA
(no CUDA)						

NA, Not Applicable.

There are three reasons why the split performance of the Jetson Nano is inferior to that of the raspberry pie 4B. First, because of the memory problem, more virtual memory needs to be configured when running programs, but the speed of virtual memory is far lower than that of running memory. The second is the CUDA problem. When running the program, the timer will timeout. The third problem is the processor. The processor model used by the Jetson Nano is Cortex-A57, which lags behind the Cortex-A72 processor of the Raspberry pie 4B. Therefore, in actual use, the performance of raspberry pie 4B is better than that of the Jetson Nano. Theoretically, the performance of the Jetson TX2 is the best (Süzen et al., 2020), but its cost is high, so it is not considered in this paper.

### 4.4 Outdoor deployment prospect

**Figure 10:**
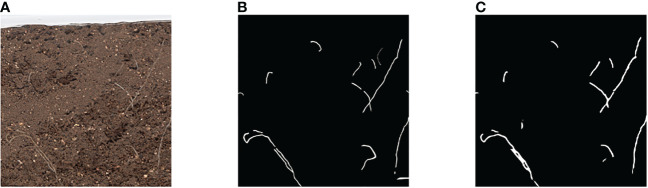
Segmentation result of root image with darker soil color **(A)** Original image **(B)** Ground Truth **(C)** Segmentation result.

Because soil color is dark and soil contains more impurities in the case of natural cultivation of plants, this paper tested the image segmentation of deep soil color and obscure root, as shown in **Figure 10**, the network designed in this paper can still be segmented. The results show that the root segmentation network designed in this paper can carry out accurate identification of *in situ* roots in various situations, and because of the portability and mobility of raspberry pie, it can be deployed outdoors in the field for experiments. The schematic diagram of field experiments is shown in **Figure 1B**. At the same time, raspberry pie can be used as a continuous working platform for image segmentation. Compared with GPU as the control, the Raspberry pie has lower power consumption, saves GPU, and enables GPU to perform more computationally demanding tasks.

In the future, this paper will consider adding Nvidia neural computing stick to raspberry pie, which can theoretically improve the segmentation speed of raspberry pie. For outdoor experiments, considering the limited storage capacity of the SD card used by Raspberry Pie, consider adding cloud storage in the future, and upload the collected images obtained by the Raspberry Pie control scanner and the identification images processed by the root segmentation network to the cloud synchronously. This can not only save the limited storage space of Raspberry Pie, but also download scanning and processing images directly from the cloud, replacing the transmission of removable storage devices, improving the work efficiency, and laying a foundation for the development of high-throughput outdoor root phenotype research.

At present, image labeling and training are classified into two categories: root and non-root. When performing root phenotypic analysis, only the whole picture can be analyzed, and the taproot and lateral root cannot be analyzed separately. In the future, this paper will consider using transfer learning to update the categories of taproot and lateral root on the basis of the current situation, which will have more agronomic significance.

In the process of root segmentation, small particles in the soil will have an impact on the segmentation results, resulting in root breakage in the segmentation results. The future goal of our experimental group is to reconstruct the root system by generating an antagonistic network and analyze the reconstructed root system.

## 5 Conclusion

This paper proposes a method to deploy semantic segmentation model to edge devices, and improves DeeplabV3+ model to segment image edges better. At the same time, we propose an image segmentation strategy which can save time and has both image details and context information. The improved DeeplabV3+ deployment in Raspberry Pie 4B shows good performance. Compared with the deployment of the split network on the GPU platform, the cost of deployment in Raspberry Pie is as low as $247, and the power consumption of 8-hour acquisition and segmentation is as low as 0.051kWh. Considering the time and cost, the accuracy of the improved model is 91.19%, and the errors of the root length and diameter are 0.669% and 1.003% respectively. The effect is similar to that of the GPU, and it is more portable than computers. It can be deployed outdoors for field experiment analysis. It can be seen that if time permits, edge devices can replace laptops to complete batch collection and segmentation of plant root images. In this paper, based on the edge equipment, the segmentation of root phenotype is effectively explored, which provides a favorable basis for the study of root phenotype from the experimental environment to the field and outdoors.

## Data availability statement

Publicly available datasets were analyzed in this study. This data can be found here: https://github.com/jiwd123/imporved_deeplabv3plus.

## Author contributions

LZ, LL, and NW initiated and designed the research. QY, HT, LZ, and WZ performed the experiments and collected the data. QY wrote the code and tested the methods. QY and HT analyzed the data and wrote the manuscript. All authors contributed to the article and approved the submitted version.

## References

[B1] Abramoff M. D. Magalhães P. J. Ram S. J. (2004). Image processing with ImageJ. Biophotonics Int. 11 (7), 36–42.

[B2] Badrinarayanan V. Kendall A. Cipolla R. (2017). SegNet: A deep convolutional encoder-decoder architecture for image segmentation. IEEE Trans. Pattern Anal. Mach. Intell. 39 (12), 2481–2495. doi: 10.1109/TPAMI.2016.2644615 28060704

[B3] Bates G. H. (1937). A device for the observation of root growth in the soil. Nature 139 (3527), 966–967. doi: 10.1038/139966b0

[B4] Borisjuk L. Rolletschek H. Neuberger T. (2012). Surveying the plant’s world by magnetic resonance imaging. Plant J. 70 (1), 129–146. doi: 10.1111/j.1365-313X.2012.04927.x 22449048

[B5] Chen L. C. Zhu Y. Papandreou G. Schroff F. Adam H. (2018). Encoder-decoder with atrous separable convolution for semantic image segmentation. In Proceedings of the Computer Vision - ECCV 2018. ECCV 2018. Lecture Notes in Computer Science Ferrari V. Hebert M. Sminchisescu C. Weiss Y. Eds. (Cham: Springer), Vol. 11211. doi: 10.1007/978-3-030-01234-2_49

[B6] Cseresnyés I. Kelemen B. Takács T. Füzy A. Kovács R. Megyeri M. . (2021). Electrical capacitance versus minirhizotron technique: A study of root dynamics in wheat–pea intercrops. Plants 10 (10), 1991. doi: 10.3390/plants10101991 34685800 PMC8540429

[B7] Das A. Schneider H. Burridge J. Ascanio A. K. M. Wojciechowski T. Topp C. N. . (2015). Digital imaging of root traits (DIRT): A high-throughput computing and collaboration platform for field-based root phenomics. Plant Methods 11 (1), 51. doi: 10.1186/s13007-015-0093-3 26535051 PMC4630929

[B8] Dobrescu A. Scorza L. C. T. Tsaftaris S. A. McCormick A. J. (2017). A “Do-It-Yourself” phenotyping system: Measuring growth and morphology throughout the diel cycle in rosette shaped plants. Plant Methods 13 (1), 95. doi: 10.1186/s13007-017-0247-6 29151842 PMC5678596

[B9] Ferreira T. R. Pires L. F. Reichardt K. (2022). 4D X-ray computed tomography in soil science: an overview and future perspectives at Mogno/Sirius. Braz. J. Phys. 52 (2), 33. doi: 10.1007/s13538-021-01043-x

[B10] Galkovskyi T. Mileyko Y. Bucksch A. Moore B. Symonova O. Price C. A. . (2012). GiA roots: software for the high throughput analysis of plant root system architecture. BMC Plant Biol. 12 (1), 116. doi: 10.1186/1471-2229-12-116 22834569 PMC3444351

[B11] Hammac W. A. Pan W. L. Bolton R. P. Koenig R. T. (2011). High resolution imaging to assess oilseed species’ root hair responses to soil water stress. Plant Soil 339 (1), 125–135. doi: 10.1007/s11104-010-0335-0

[B12] Hinsinger P. Brauman A. Devau N. Gérard F. Jourdan C. Laclau J.-P. . (2011). Acquisition of phosphorus and other poorly mobile nutrients by roots. where do plant nutrition models fail? Plant Soil 348 (1), 29. doi: 10.1007/s11104-011-0903-y

[B13] Horn J. Zhao Y. Wandel N. Landl M. Schnepf A. Behnke S. (2021). “Robust skeletonization for plant root structure reconstruction from MRI,” in 2020 25th International Conference on Pattern Recognition (ICPR). (Milan, Italy: IEEE), 10689–10696. doi: 10.1109/ICPR48806.2021.9413045

[B14] Jahnke S. Menzel M. I. Van Dusschoten D. Roeb G. W. Bühler J. Minwuyelet S. . (2009). Combined MRI–PET dissects dynamic changes in plant structures and functions. Plant J. 59 (4), 634–644. doi: 10.1111/j.1365-313X.2009.03888.x 19392708

[B15] Jia K. A. Llbc D. Fz E. Chen S. A. Nan W. Ls A. (2021). Semantic segmentation model of cotton roots *in-situ* image based on attention mechanism. Comput. Electron. Agric. 189, 106370. doi: 10.1016/j.compag.2021.106370

[B16] Jolles J. W. (2021). Broad-scale applications of the raspberry pi: A review and guide for biologists. Methods Ecol. Evolution 12 (9), 1562–1579. doi: 10.1111/2041-210X.13652

[B17] Le Bot J. Serra V. Fabre J. Draye X. Adamowicz S. Pagès L. (2010). DART: A software to analyse root system architecture and development from captured images. Plant Soil 326 (1), 261–273. doi: 10.1007/s11104-009-0005-2

[B18] Liu X. Gu H. Han J. Jiang H. Duan S. (2020b). Research progress of ground penetrating radar and electrical capacitance for in-situ non-destructive measurement of crop roots. Trans. Chin. Soc. Agric. Eng. 36 (20), 226–237.

[B19] Liu W. Shu Y. Tang X. Liu J. (2020a). Remote sensing image segmentation using dual attention mechanism Deeplabv3+ algorithm. Trop. Geogr. 40 (2), 303–313. doi: 10.13284/j.cnki.rddl.003229

[B20] Lynch J. P. (2013). Steep, cheap and deep: an ideotype to optimize water and n acquisition by maize root systems. Ann. Bot. 112 (2), 347–357. doi: 10.1093/aob/mcs293 23328767 PMC3698384

[B21] Lynch J. P. Wojciechowski T. (2015). Opportunities and challenges in the subsoil: pathways to deeper rooted crops. J. Exp. Bot. 66 (8), 2199–2210. doi: 10.1093/jxb/eru508 25582451 PMC4986715

[B22] Metzner R. Eggert A. van Dusschoten D. Pflugfelder D. Gerth S. Schurr U. . (2015). Direct comparison of MRI and X-ray CT technologies for 3D imaging of root systems in soil: Potential and challenges for root trait quantification. Plant Methods 11 (1), 17. doi: 10.1186/s13007-015-0060-z 25774207 PMC4359488

[B23] Mohamed A. Monnier Y. Mao Z. Lobet G. Maeght J.-L. Ramel M. . (2017). An evaluation of inexpensive methods for root image acquisition when using rhizotrons. Plant Methods 13 (1), 11. doi: 10.1186/s13007-017-0160-z 28286541 PMC5341412

[B24] Nahar K. Pan W. L. (2019). High resolution *in situ* rhizosphere imaging of root growth dynamics in oilseed castor plant (Ricinus communis l.) using digital scanners. Modeling Earth Syst. Environ. 5 (3), 781–792. doi: 10.1007/s40808-018-0564-4

[B25] Nakahata R. Osawa A. (2017). Fine root dynamics after soil disturbance evaluated with a root scanner method. Plant Soil 419 (1), 467–487. doi: 10.1007/s11104-017-3361-3

[B26] Narisetti N. Henke M. Seiler C. Junker A. Ostermann J. Altmann T. . (2021). Fully-automated root image analysis (faRIA). Sci. Rep. 11 (1), 16047. doi: 10.1038/s41598-021-95480-y 34362967 PMC8346561

[B27] Nielsen K. L. Eshel A. Lynch J. P. (2001). The effect of phosphorus availability on the carbon economy of contrasting common bean (Phaseolus vulgaris l.) genotypes. J. Exp. Bot. 52 (355), 329–339. doi: 10.1093/jexbot/52.355.329 11283178

[B28] Paez-Garcia A. Motes C. M. Scheible W.-R. Chen R. Blancaflor E. B. Monteros M. J. (2015). Root traits and phenotyping strategies for plant improvement. Plants 4 (2), 334–355. doi: 10.3390/plants4020334 27135332 PMC4844329

[B29] Park J. Seo D. Kim K. W. (2020). X-Ray computed tomography of severed root wounds of prunus serrulata and zelkova serrata. For. Pathol. 50 (4), e12622. doi: 10.1111/efp.12622

[B30] Pflugfelder D. Kochs J. Koller R. Jahnke S. Mohl C. Pariyar S. . (2021). The root system architecture of wheat establishing in soil is associated with varying elongation rates of seminal roots: Quantification using 4D magnetic resonance imaging. J. Exp. Bot. 73 (7), 2050–2060. doi: 10.1093/jxb/erab551 34918078

[B31] Pierret A. Gonkhamdee S. Jourdan C. Maeght J.-L. (2013). IJ_Rhizo: an open-source software to measure scanned images of root samples. Plant Soil 373 (1), 531–539. doi: 10.1007/s11104-013-1795-9

[B32] Pound M. P. French A. P. Atkinson J. A. Wells D. M. Bennett M. J. Pridmore T. (2013). RootNav: Navigating images of complex root architectures. Plant Physiol. 162 (4), 1802–1814. doi: 10.1104/pp.113.221531 23766367 PMC3729762

[B33] Rajurkar A. B. McCoy S. M. Ruhter J. Mulcrone J. Freyfogle L. Leakey A. D. B. (2022). Installation and imaging of thousands of minirhizotrons to phenotype root systems of field-grown plants. Plant Methods 18 (1), 39. doi: 10.1186/s13007-022-00874-2 35346269 PMC8958774

[B34] Ren F. He X. Wei Z. Lv Y. Li M. (2019). Sematic segmentation based on DeepLabV3+ and superpixel optimization. Optics Precis. Eng. 27 (12), 2722–2729. (in Chinese).

[B35] Ronneberger O. Fischer P. Brox T. (2015). “U-Net: Convolutional networks for biomedical image segmentation” in Lecture Notes in Computer Science Eds Navab N. Hornegger J. Wells W. Frangi A. (Cham: Springer) 9351, 234–241. doi: 10.1007/978-3-319-24574-4_28

[B36] Schneider H. M. Postma J. A. Kochs J. Pflugfelder D. Lynch J. P. van Dusschoten D. (2020). Spatio-temporal variation in water uptake in seminal and nodal root systems of barley plants grown in soil. Front. Plant Sci. 11. doi: 10.3389/fpls.2020.01247 PMC7438553 32903494

[B37] Scotson C. P. van Veelen A. Williams K. A. Koebernick N. McKay Fletcher D. Roose T. (2021). Developing a system for *in vivo* imaging of maize roots containing iodinated contrast media in soil using synchrotron XCT and XRF. Plant Soil 460 (1), 647–665. doi: 10.1007/s11104-020-04784-x 34720206 PMC8550435

[B38] Seethepalli A. Dhakal K. Griffiths M. Guo H. Freschet G. T. York L. M. (2021). RhizoVision explorer: Open-source software for root image analysis and measurement standardization. AoB Plants 13 (6), plab056. doi: 10.1093/aobpla/plab056 34804466 PMC8598384

[B39] Seethepalli A. Guo H. Liu X. Griffiths M. Almtarfi H. Li Z. . (2020). RhizoVision crown: An integrated hardware and software platform for root crown phenotyping. Plant Phenomics 2020, 3074916. doi: 10.34133/2020/3074916 33313547 PMC7706346

[B40] Seidenthal K. Panjvani K. Chandnani R. Kochian L. Eramian M. (2022). Iterative image segmentation of plant roots for high-throughput phenotyping. Sci. Rep. 12 (1), 16563. doi: 10.1038/s41598-022-19754-9 36195610 PMC9532414

[B41] Shan J. Tao D. (1992). Overseas researches on tree fine root. Chin. J. Ecol. 11 (4), 46–49.

[B42] Shen C. Liu L. Zhu L. Kang J. Shao L. (2020). High-throughput *in situ* root image segmentation based on the improved DeepLabv3+ method. Front. Plant Sci. 11, 576791. doi: 10.3389/fpls.2020.576791 33193519 PMC7604297

[B43] Smith A. G. Han E. Petersen J. Olsen N. A. F. Giese C. Athmann M. . (2022). RootPainter: deep learning segmentation of biological images with corrective annotation. New Phytol. 236 (2), 774–791. doi: 10.1111/nph.18387 35851958 PMC9804377

[B44] Sun K. Xiao B. Liu D. Wang J. (2019). “Deep high-resolution representation learning for human pose estimation,” in 2019 IEEE/CVF Conference on Computer Vision and Pattern Recognition (CVPR). (Long Beach, CA, USA: IEEE), 5686–5696. doi: 10.1109/CVPR.2019.00584

[B45] Süzen A. A. Duman B. Şen B. (2020). “Benchmark analysis of jetson TX2, jetson nano and raspberry PI using deep-CNN,” in 2020 International Congress on Human-Computer Interaction, Optimization and Robotic Applications (HORA). (Ankara, Turkey: IEEE), 1–5. doi: 10.1109/HORA49412.2020.9152915

[B46] Tausen M. Clausen M. Moeskjær S. Shihavuddin A. Dahl A. B. Janss L. . (2020). Greenotyper: Image-based plant phenotyping using distributed computing and deep learning. Front. Plant Sci. 11. doi: 10.3389/fpls.2020.01181 PMC7427585 32849731

[B47] Valle B. Simonneau T. Boulord R. Sourd F. Frisson T. Ryckewaert M. . (2017). PYM: A new, affordable, image-based method using a raspberry pi to phenotype plant leaf area in a wide diversity of environments. Plant Methods 13 (1), 98. doi: 10.1186/s13007-017-0248-5 29151844 PMC5678554

[B48] Wang T. Rostamza M. Song Z. Wang L. McNickle G. Iyer-Pascuzzi A. S. . (2019). SegRoot: A high throughput segmentation method for root image analysis. Comput. Electron. Agric. 162, 845–854. doi: 10.1016/j.compag.2019.05.017

[B49] Woo S. Park J. Lee J.-Y. Kweon I. S. (2018). “Cbam: Convolutional block attention module,” in Proceedings of the European conference on computer vision (ECCV). (Cham: Springer), 3–19. doi: 10.1007/978-3-030-01234-2_1

[B50] Xiao S. Liu L. Zhang Y. Sun H. Bai Z. Zhang K. . (2020). Review on new methods of *in situ* observation of plant micro-roots and interpretation of root images. J. Plant Nutr. Fertilizers 26 (2), 370–385. (in Chinese).

[B51] Yasrab R. Atkinson J. A. Wells D. M. French A. P. Pridmore T. P. Pound M. P. (2019). RootNav 2.0: Deep learning for automatic navigation of complex plant root architectures. Gigascience 8 (11), giz123. doi: 10.1093/gigascience/giz123 31702012 PMC6839032

[B52] Zhao H. Shi J. Qi X. Wang X. Jia J. (2017). “Pyramid scene parsing network,” in 2017 IEEE Conference on Computer Vision and Pattern Recognition (CVPR). (Honolulu, HI, USA: IEEE), 6230–6239. doi: 10.1109/CVPR.2017.660

[B53] Zhao H. Wang N. Sun H. Zhu L. Zhang K. Zhang Y. . (2022). RhizoPot platform: A high-throughput *in situ* root phenotyping platform with integrated hardware and software. Front. Plant Sci. 13. doi: 10.3389/fpls.2022.1004904 PMC9558169 36247541

