# Supplementary figures and images for: A method of cotton root segmentation based on edge devices
Source: Front Plant Sci. 2023 Feb 17;14:1122833. doi: 10.3389/fpls.2023.1122833 (PMC9982017; doi:10.3389/fpls.2023.1122833)

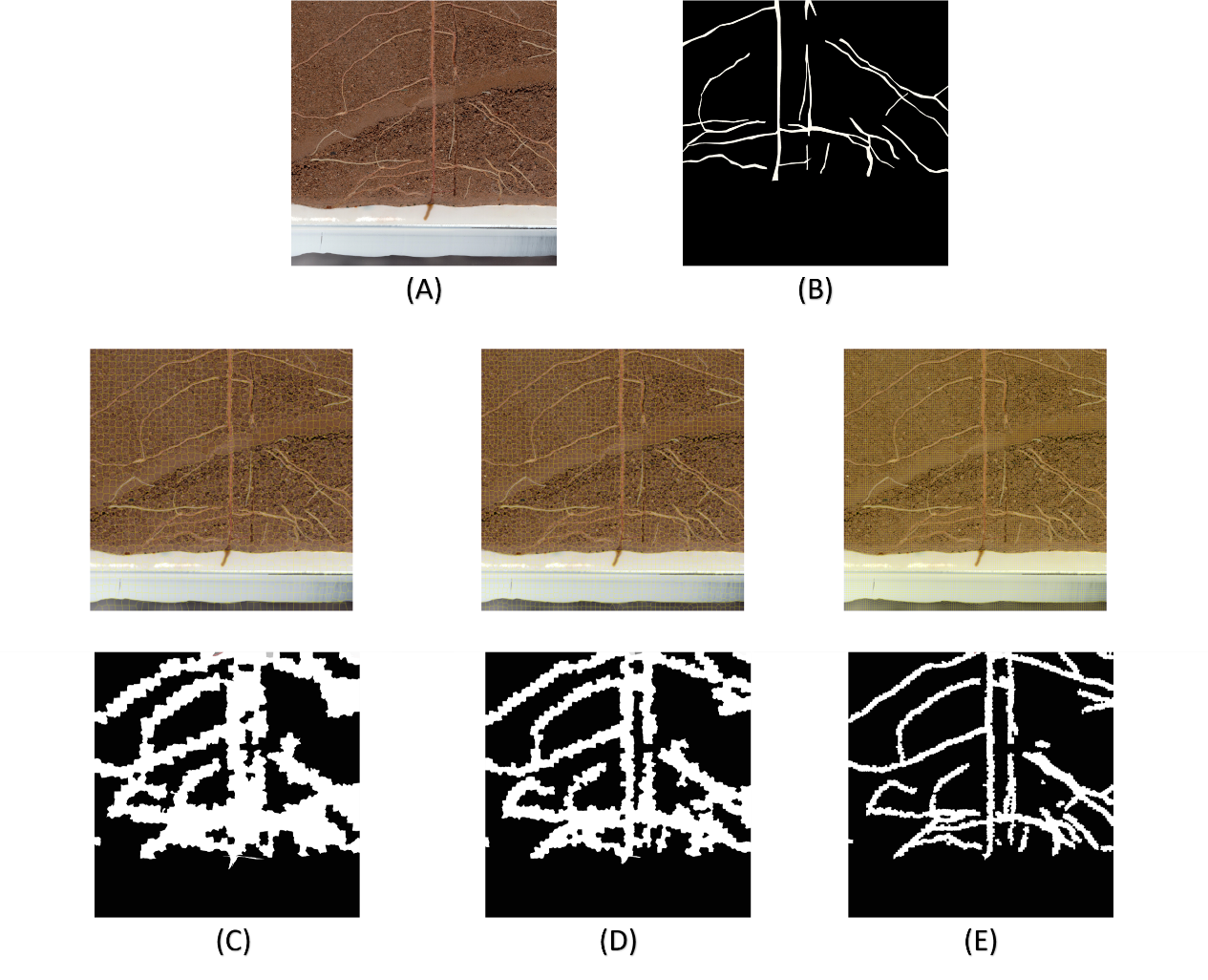

Supplement: Supplementary file 1 [file Image_1.tif]
